# Evaluation of disaster safe education unit programme implementation in Mt. Merapi using the pressure state response approach

**DOI:** 10.4102/jamba.v16i1.1769

**Published:** 2024-11-29

**Authors:** Puspita I. Wardhani, Muhammad Musiyam, Yunus A. Wibowo, Aries Dwi W. Rahmadana, Sri Utami, Edwin Maulana

**Affiliations:** 1 Department of Geography Education, Faculty of Teacher Training and Education, Universitas Muhammadiyah Surakarta, Sukoharjo, Indonesia; 2 CV Geo Art Science, Sleman, Indonesia; 3 Department of Geographic Information System, Universitas Maha Karya Asia, Sleman, Indonesia; 4 Centre for Disaster Studies, Universitas Gadjah Mada, Sleman, Indonesia; 5 Department of Environmental Science, The Graduate School, Universitas Gadjah Mada, Sleman, Indonesia

**Keywords:** safe school, SPAB, PSR, Merapi, evaluation, implementation, disaster, eruption

## Abstract

Disaster Safe Education Unit (SPAB), also known globally as Comprehensive Safe School (CSS), aims to improve school residents’ resilience. Three pillars of SPAB have been established, but their implementation, to date, has not shown satisfactory results. This study aims to evaluate the implementation of the SPAB programme in the disaster-prone area (KRB) of Merapi Vulcano in Sleman, Yogyakarta, Indonesia. Data collection was conducted using a field survey combined with in-depth interviews. Overall, the study focussed on the 32 schools in the KRB Merapi. The SPAB implementation was evaluated by considering the Pressure-State-Response (PSR) indicator. The study results showed that not all schools in KRB Merapi implemented the SPAB programme. Safe schools have not fully implemented the three pillars of SPAB. Pillar 3 had the highest rate (96.40%) of implementation in safe schools, whereas Pillar 1 had the lowest (54.5%). Legality, funding and human resources are the critical plausible explanations for why the SPAB pillars have not been implemented well. These problems affect school infrastructure, the capacity of educators, consistency and school motivation in implementing SPAB programme.

**Contribution:**

The response to overcome these issues is strengthening regulations related to SPAB implementation. Strong regulations will provide space for funding components to increase the capacity of school residents, improve infrastructure, as well as increase school motivation. Stakeholders can utilise these findings to formulate more robust regulatory formulations for implementing SPAB in other KRB zones with similar typologies.

## Introduction

Volcanic eruptions are natural calamities that can dramatically inflict massive losses. Volcanic eruptions threaten environmental sustainability and endanger human life around the volcano (López-Saavedra & Martí 2023; Massaro et al. 2023; Thouret et al. 2023). Pyroclastic material can cause death, damage public facilities and infrastructure, and disrupt life and livelihood (Massaro et al. 2023; Weir et al. 2024). Volcanic eruptions can also affect climate conditions, as happened in Samalas (1257 CE) and Tambora (1815 CE) (Malawani et al. 2021). Several initiatives have been taken by Indonesian government to minimise the impact of eruptions, including increasing community capacity through education (Andreastuti et al. 2023; Thouret et al. 2023).

Capacity building is an educational attempt to mitigate the effects of disasters. The Hyogo Framework for Action (HFA) places education as one of the critical priorities in minimising the threat of disasters (Amri et al. 2022; Roopnarine et al. 2021). Schools, especially students, are considered the most appropriate place to increase community capacity (Amri et al. 2022). Agarwal et al. (2023) developed a disaster education framework in schools and found that schools have the physical and human components that interact to provide continuous instructions in a secure environment. Collaboration between teachers and students can indirectly facilitate the dissemination of disaster information (Gokmenoglu et al. 2021). Furthermore, students can contribute to boost community resilience, at least at the household level.

As a country with high vulnerability to multi-disaster threats, the Indonesian government plays an active role in increasing community capacity through various initiatives. The Disaster Safe Education Unit (SPAB) programme in Indonesia is one such initiative that aims to provide disaster education and a safe learning environment for children affected by natural or social disasters. The Regulation of the Secretary General of the Ministry of Education, Culture, Research, and Technology (Persekjen Kemenristek) Number 6 of 2023 stipulates the Technical Instructions for the Implementation of the SPAB. The regulation mandates the implementation of SPAB to increase the resilience of students and schools in disaster-prone area (KRB) zones (Amri et al. 2022; Ca & Anam 2024). Globally, SPAB is known as a comprehensive safe school (CSS), which is a comprehensive strategy for a safe learning environment for teachers, students and school staff (Iqbal & Nauman 2024).

Three main pillars underlie the implementation of SPAB: (1) Safer Learning Facilities; (2) Security of Educational Units and Management of Educational Continuity; and (3) Risk Reduction and Resilience Education. These three pillars align with the mandate of the Global Alliance for Disaster Risk Reduction and Resilience in the Education Sector (GADRRRES), establishing the three school safety pillars. Interestingly, even though Indonesia is vulnerable to multi-disaster threats, implementing SPAB is not mandatory (Desilia, Lassa & Oktari 2023). This phenomenon causes not all schools in KRB zones to implement the SPAB framework. This is an intriguing topic to investigate further to pinpoint multiple challenges with applying SPAB framework.

Merapi (2968 m) is the most active volcano in Indonesia, located on the border of Yogyakarta and Magelang, Indonesia. For approximately 500 years, at least 73 significant eruptions have been recorded (Chan, Konstantinou & Blackett 2021). Interestingly, the Merapi environment is relatively densely populated, hence many people are exposed to the risk of Merapi eruptions (Chan et al. 2021; Wigati et al. 2023). One of the most dramatic eruptions was the 2010 eruption, which caused at least 17 deaths (Wigati et al. 2023). In addition, 4874 people experienced psychiatric problems, and 1705 people were injured (Brotopuspito et al. 2011). The 2010 eruption blew a column of ash up to a height of 17 km, and its pyroclastic material reached an area up to 16 km from the peak of Merapi (Chasanah & Sakakibara 2022). The last eruption occurred in 2018, with a volcanic eruption index reaching 3 (Chan et al. 2021). In spite of the risks that have taken lives, the area around Merapi remains densely populated and serves as a re-migration site for evacuated people (Muir et al. 2020). Increasing community capacity, especially at the school level, needs to be improved to increase awareness of the risk of a Merapi eruption.

The Merapi environment poses a high risk because of short recurrence time (less than 10 years) of the volcanic eruptions. This risk must be addressed adequately by boosting population capacity, which includes implementing the SPAB framework. Although it is not mandatory, several schools in Yogyakarta have implemented the SPAB scheme (Cabatay & Gonzales 2024; Desilia et al. 2023). Several schools face difficulties in applying the SPAB framework because of the complicated nature of the SPAB pillars. This study aims to evaluate the implementation of the SPAB pillars in elementary to high school education in the KRB zones of Mount Merapi.

## Research methods and design

**FIGURE 1:**
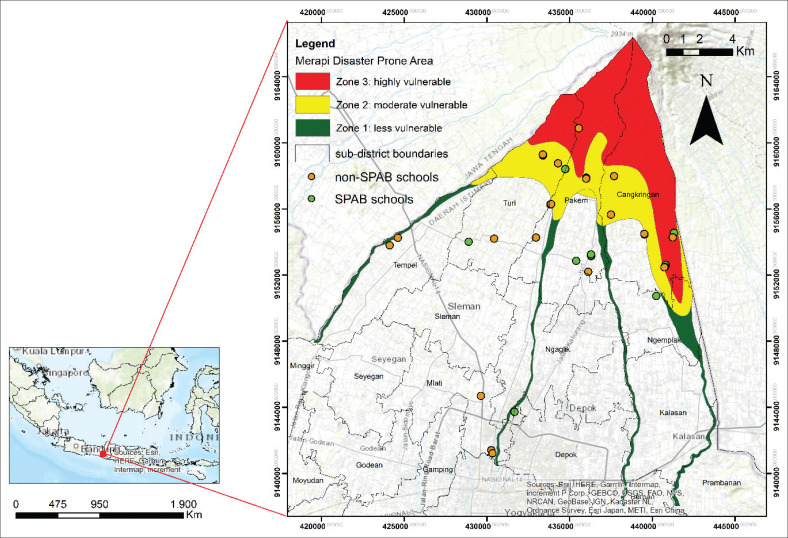
Study site.

This study was conducted in several private schools in the Merapi KRB zone of the Yogyakarta Special Region (DIY), Indonesia. According to the Minister of Energy and Mineral Resources (ESDM) Regulation No. 11 of 2016, KRB Merapi is classified into three zones: KRB I (high vulnerability), KRB II (medium vulnerability), and KRB III (low vulnerability). Most of the schools are in the KRB II and KRB I zones (see Figure 1). KRB III tends not to have schools because the area is often hit by hot clouds, lava flows, volcanic bombs, toxic gases and rock falls (incandescent). Community activities are not so prevalent in KRB III zone because it is not used for settlements (BPBD DIY 2023).

The pre-field process involved selecting multiple schools as research samples. The analysis began with a multi-layer imposed between the Merapi KRB Map and school location data. The Merapi KRB Map presents the zoning of the Merapi disaster vulnerability level obtained from the Center for Volcanology and Geological Disaster Mitigation (PVMBG) of ESDM. Tabular school data were gathered from the DIY Education Bureau and then converted into spatial data to see the distribution of schools. The study was conducted at both SPAB-certified and non-SPAB schools. Overall, 32 educational units were examined, ranging from preschool (5 schools) to kindergarten (12 schools), elementary school (6 schools), junior high (3 schools), and senior high (6 schools). Schools were chosen based on their position in the KRB zone or outside the KRB zone, both of which could be affected by the Merapi eruption. The selection of school samples from the three KRB zones was intended to determine the variation in the level of awareness and implementation of SPAB across schools with varying levels of exposure to the Merapi eruption.

Field surveys were conducted to collect information about the condition of schools and their surroundings that support the implementation of safe schools. Surveys were carried out to determine the school’s physical structure, disaster preparedness resources and proximity to the Merapi hazard source. In addition, the purpose of this phase was to collect data pertaining to SPAB Pillar 1, which is safe school facilities. Safe school facilities have buildings, contents and surrounding yards that meet safety and security requirements, especially in dealing with volcanic hazards. In this scenario, the visible elements include the school’s proximity to rivers, lava flood paths and other secondary hazards. Facilities include School Health Units, evacuation points, evacuation routes and warning sirens.

In-depth interviews were conducted with key figures in charge of school disaster preparedness, including the principal, student team and teachers appointed as in-charge of the Safe School programme. In general, in-depth interviews contain information on implementing the SPAB pillars and their obstacles. Specifically, Table 1 shows the indicators used to evaluate SPAB implementation and is presented as a structured questionnaire. Furthermore, the results of the interviews were analysed to display data on the achievement of the SPAB pillars. Quantitative graphs were made to analyse the interview data. The achievements of the pillars were analysed based on the safe school and non-SPAB school groups to determine the school’s readiness for the safe school programme. It is important to note that the interview process was expanded to include a deeper examination of the school’s progress toward becoming SPAB, specifically for institutions with SPAB predicate. In non-SPAB schools, information exploration was undertaken to determine whether the school planned on participating in the SPAB programme when it was appointed. Considering the interview process, the reasons behind implementing the less-than-ideal pillar might be revealed. A total of 32 respondents were involved, distributed throughout the three KRB locations.

**TABLE 1:** Disaster safe education unit pillar indicators.

Pillar	Indicators
Pillar 1 (Safe school facilities)	School construction considering the risk of Merapi disaster threat
	Disaster mitigation facilities owned by the school
	Regular maintenance of disaster equipment
	Assessment of the condition and strength of school infrastructure
Pillar 2 (Disaster Management in Schools)	Disaster Risk Assessment in Educational Units
	Disaster preparedness team
	SPAB Partnership
	SPAB related policies
	Action plan preparation
Pillar 3 (Education, Prevention and Disaster Risk Reduction)	SPAB programme socialisation
	Disaster Risk Reduction (DRR) material integration into the curriculum
	Extracurricular activities that support disaster preparedness efforts
	DRR training for teachers and disaster preparedness teams
	Implementation of disaster emergency response simulations

*Source*: Badan Nasional Penanggulangan Bencana (BNPB), n.d., *Monev Satuan Pendidikan Aman Bencana*, viewed 17 July 2024, from https://inarisk2.bnpb.go.id/spab/ (modified)

SPAB, disaster safe education unit.

Identifying problems in SPAB implementation is analysed using the Pressure-State-Response (PSR) indicator. The PSR indicator was developed by the Organisation for Economic Co-operation and Development (OECD) and is widely used to assess environmental issues and as a tool for compiling reports (Wang et al. 2021). Furthermore, the PSR indicator is also used to evaluate human pressure on the environment so that recommendations can be made regarding political responses to achieve ideal conditions (Levrel et al. 2009). In order to determine the obstacles and to develop the possibility for SPAB implementation, the PSR indicator was adopted and changed. Pressure explains external and internal factors that cause the need to strengthen SPAB, while state refers to information related to school awareness and readiness for SPAB implementation. The response is to formulate efforts or strategies to strengthen the SPAB pillars. Exploratory descriptive analysis explains the phenomena that occur in SPAB implementation problems in the Merapi area.

### Ethical considerations

The Head of Muhammadiyah Regional, D.I. Yogyakarta gave permission to lecturers from the Department of Geography Education Program, Faculty of Teacher Training and Education at the Universitas Muhammadiyah Surakarta (UMS), to conduct research at Muhammadiyah schools in Sleman Regency to gather the data needed for the research (No. 167/II.4/F/2024).

## Results

### Descriptive statistics of educational units in the Merapi disaster-prone area zone

**FIGURE 2:**
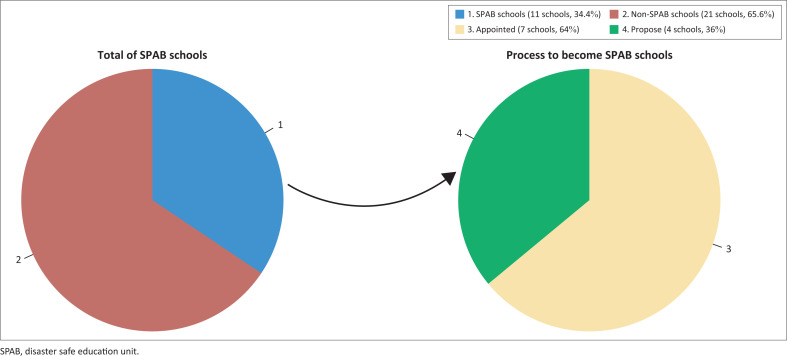
Number of SPAB and non-SPAB Schools.

The study results showed that out of the 32 private schools surveyed, 34.4% or 11 schools had become SPAB. Most of the schools designated as SPAB have been affected by the eruption of Merapi. However, some schools in the KRB Merapi area have been designated as SPAB because of the threat of other disasters such as hurricanes. These schools are spread across KRB zones I, II, and III. The KRB III area has two SPAB schools at the elementary and senior high school levels. The school levels in KRB Merapi that have become SPAB are elementary school, junior high school, and senior high school, while preschool and kindergarten schools have not yet become SPAB (Figure 2). This needs to be specially taken note of, considering that many preschools and kindergartens are in the KRB III zone and vulnerable to volcanic disasters. Children in early childhood require special treatment in education about disasters. The practice hitherto is that schools rely on the parents of students or the village alert team in case of an emergency.

The process to become SPAB was carried out once but was divided into several years. The SPAB programme in KRB Merapi began to be promoted in 2011, which was previously still called the Disaster Preparedness School (SSB). Merapi’s eruption in 2010 provided enormous momentum, raising awareness among various parties regarding the significance of disaster education in schools. The process of schools becoming SPABs was mainly handled by the Regional Disaster Management Agency (BPDB), with the primary considerations being the location of the school and the school’s experience in being affected by the Merapi eruption. Most of the SPAB schools studied were inaugurated after the Merapi eruption. However, some were inaugurated recently, namely in 2020 and 2022. This shows the need for the role of the SPAB secretariat to encourage and assist so that more schools can become SPABs.

### Implementation of disaster safe education unit pillars in the Merapi disaster-prone area zone

The implementation of the SPAB programme is outlined in a framework containing three pillars that must be met. Assessment of the SPAB pillar implementation in safe schools is essential to see each school’s SPAB programme’s success level. Assessment of the SPAB pillars in non-SPAB schools is also essential to see the readiness of the school if it is officially designated as an SPAB. Several pillar indicators are activities that schools have developed to reduce the impact of disasters without the need for a safe school predicate. For example, scouting extracurricular activities at the elementary and secondary school levels are indirectly associated with disaster education in schools. The conditions and processes in place at the school level determine which pillars are applied. Table 1 shows the indicators used in assessing the achievement of the SPAB pillars.

**FIGURE 3:**
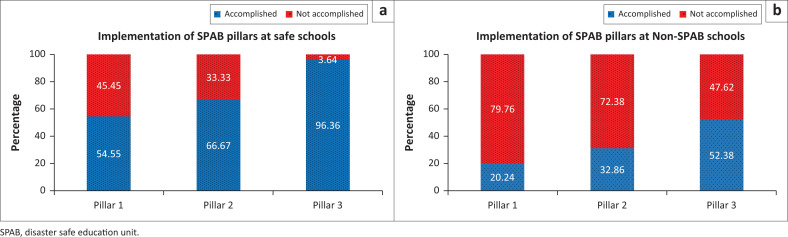
Comparison of: a) Implementation of SPAB pillars at safe schools; and b) Implementation of SPAB pillars at Non-SPAB schools.

Safe schools have a higher percentage of pillar implementation than non-SPAB schools, with a percentage difference that is two times (Figure 3). The implementation of Pillar 3 reached 96.4% in safe schools, while in non-SPAB schools it was only 53.3%. Pillar 3 is the most accessible pillar to implement, including in non-SPAB schools. Pillar 3 contains disaster education, prevention and risk reduction. Regarding disaster education, both safe and non-SPAB schools generally include disaster education in specific subjects such as natural sciences or local regional knowledge. Furthermore, this highly depends on the teacher’s initiative because no standardised syllabus for specific material exists. Disaster simulations need to be used to further optimise the provision of disaster knowledge in the classroom. Schools appointed as safe schools are more likely to undertake simulations regularly. All safe schools surveyed have conducted disaster simulations.

**FIGURE 4:**
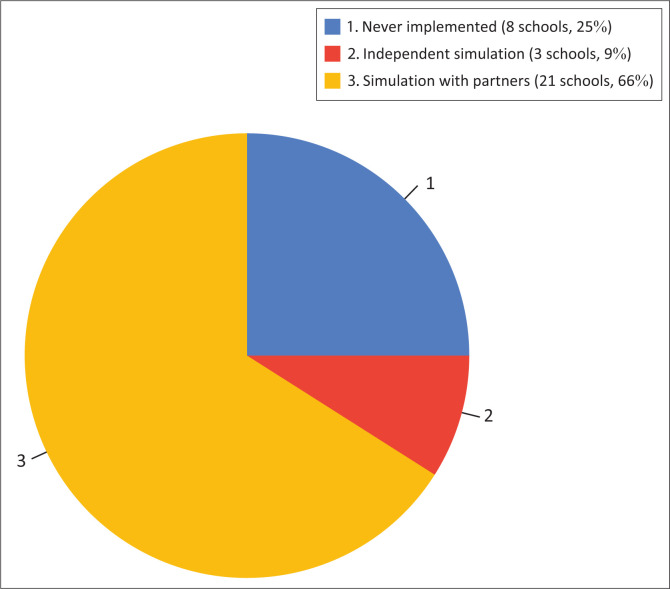
Disaster simulation activities.

The simulation is primarily implemented through collaboration with partners. Stakeholders who help implement disaster simulations in the Merapi KRB Area are Badan Penanggulangan Bencana Daerah (BPBD) and Disaster Response Units owned by religious foundations. At least, the simulation is carried out once a year. Many schools have not been able to carry out disaster simulations independently. Based on the 32 schools surveyed, only 9% could conduct disaster simulations independently, while 25% had never conducted a disaster simulation (Figure 4). The biggest reason for schools’ inability to conduct disaster simulations is the lack of facilities, infrastructure and competent facilitators. Some teachers and school-level disaster preparedness teams have received disaster training, but not all schools have received comprehensive materials to conduct independent disaster simulations. As many as 81% of the teachers and/or disaster preparedness teams in safe schools have received training, while for non-SPAB schools, it is only 47%.

Typically, the pillar with the highest implementation is Pillar 3, followed by Pillar 2, and the last being Pillar 1. The pattern is the same for both safe and non-SPAB schools. Pillar 1 (safe school facilities) is the most difficult pillar to implement because it requires partnership assistance to procure disaster facilities and infrastructure. There are still few schools that are aware of allocating a particular budget for disaster preparedness. Pillar 2 (Disaster Management in Schools) has a reasonably high implementation in safe schools but is still low in non-SPAB schools because no regulations serve as a basis for implementing it. This is a sign that the SPAB school predicate significantly impacts disaster preparedness efforts in schools.

### Pressure-state-response approach to identifying the disaster safe education unit implementation

Pressure is broadly grouped into two types, namely external and internal pressure. External pressure in implementing SPAB includes: (1) partner absence, (2) monitoring, (3) legality, and (4) incentives. Internal pressure includes: (1) facilities, (2) funding, (3) human resources, and (4) capacity. The legality factor is the most critical component in implementing SPAB, considering that legality can impact the entire process of implementing SPAB. Interestingly, SPAB has been optional until now, and is only now becoming mandatory. This optional nature impacts school policies that cannot budget funding to support the SPAB pillars. This affects capacity, human resources and infrastructural conditions that need to be improved.

In terms of physical aspects, the pressure that occurs causes the infrastructure to support SPAB to be classified as less than ideal. Furthermore, the absence of supervision related to the implementation of SPAB causes the consistency and accountability of programme implementation to be neglected. An interesting phenomenon also occurs in the rotation of educators. Teachers, as educators, are usually involved in preparing a roadmap for implementing SPAB but are suddenly transferred to another school. This results in no leading person in the school to replace the teacher concerned. In the absence of teachers as driving figures, students’ capacity and enthusiasm to execute the SPAB pillars are poor.

**FIGURE 5:**
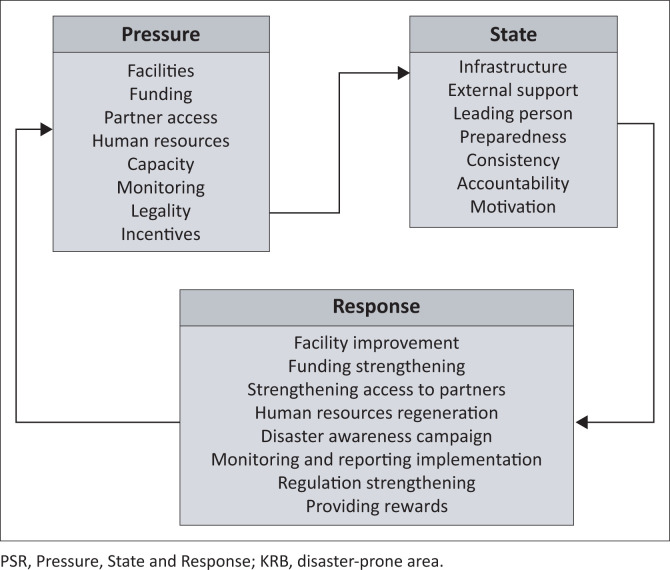
PSR analysis of the disaster safe education unit implementation business process at KRB Merapi.

The analysis shows that the response that has the most decisive influence on the implementation of SPAB is strengthening regulations. Strengthening regulations can have a domino effect on other activities that have the potential to support strengthening the SPAB pillars. Strong regulations allow schools to allocate funding to strengthen the SPAB pillars. Through substantial funding, schools can improve facilities, increase teacher capacity collectively and conduct disaster campaigns for school residents. Furthermore, strengthening regulations in government ranks allows related agencies to provide incentives to schools that have implemented SPAB. Providing incentives can increase school motivation to implement the SPAB programme. The government can also use strengthening regulations to monitor and assess the effectiveness of SPAB implementation in schools located in the Merapi KRB. The results of the PSR analysis are presented in Figure 5.

## Discussion

Disaster prevention and risk reduction education are crucial to improving community preparedness and capacity in facing a disaster. Implementing SPAB in KRB is essential in disaster risk reduction (Amri et al. 2022; Cabatay & Gonzales 2024; Iqbal & Nauman 2024). The findings show that not all schools in KRB Merapi implement the SPAB programme because of the optional nature of the programme (Amri et al. 2022; Desilia et al. 2023). Several schools that have implemented SPAB also need help implementing the SPAB pillars optimally. Desilia et al. (2023) emphasise the importance of clear regulations to integrate the three SPAB pillars properly.

Regarding the implementation of the SPAB pillars, research findings show that Pillar 3 has the highest representation in educational units in KRB Merapi. Efforts to implement Pillar 3 are carried out in several forms, such as simulations and disaster education. Interestingly, according to the study conducted by Septikasari et al. (2024), the urgency of implementing Pillar 1 is higher (2.99) than Pillar 2 (2.74) and Pillar 3 (1.56). Pillar 1, related to safe school facilities, cannot be adequately implemented because of funding constraints. Pillar 2, related to Disaster Management in Schools, has also not been fully implemented roperly. Teachers’ high workload influences this; therefore, teachers’ attention to implementing SPAB needs to be addressed (Amri et al. 2022). Furthermore, teachers’ understanding of disaster risk reduction issues also needs improvement (Amri et al. 2022; Desilia et al. 2023; Septikasari et al. 2024).

Finally, there are still gaps in implementing the SPAB pillars at the research location. This finding was confirmed by Amri et al. (2022), who stated that no systematic programme related to leadership strengthening or transformation aims to implement all SPAB pillars. Leadership strengthening programmes should be carried out integratively in schools and at the local government level. Leadership strengthening can be carried out through various activities, such as workshops or training, to properly implement the three SPAB pillars (Desilia et al. 2023; Iqbal & Nauman 2024).

## Conclusion

The implementation of the safe school programme in private schools in KRB Merapi has yet to be able to run in an integrated manner between its three pillars. This is because of the need for increased legality, funding and human resources. Among the three pillars of SPAB, the highest implementation is in Pillar 3, that is 53.5% in schools that have not implemented safe schools and 96.40% in schools that have implemented safe schools. The lowest implementation is in Pillar 1, that is 79.80% in schools that have not implemented safe schools and 45.50% in schools that have implemented safe schools.

The results of the analysis using the PSR method concluded that external pressure in the implementation of SPAB includes: (1) partner absence, (2) monitoring, (3) legality, and (4) incentives, while internal pressure includes: (1) facilities, (2) funding, (3) human resources, and (4) capacity. For the state, the most influential indicators in implementing SPAB are infrastructure and the leading person. The response indicator with the most substantial influence on the implementation of SPAB is strengthening regulations. Strengthening regulations can have a domino effect on other activities that have the potential to support strengthening the SPAB pillar.
